# An Internet-Based Intervention for Cardiovascular Disease Management Integrated With Primary Care Electronic Health Records: Mixed Methods Evaluation of Implementation Fidelity and User Engagement

**DOI:** 10.2196/25333

**Published:** 2021-04-26

**Authors:** Genevieve Coorey, David Peiris, Anish Scaria, John Mulley, Lis Neubeck, Nashid Hafiz, Julie Redfern

**Affiliations:** 1 The George Institute for Global Health, Sydney, Australia Sydney Australia; 2 School of Health Sciences Faculty of Medicine and Health University of Sydney Sydney Australia; 3 Faculty of Medicine The University of New South Wales Sydney Australia; 4 School of Health and Social Care Edinburgh Napier University Edinburgh United Kingdom; 5 Susan Wakil School for Nursing and Midwifery The University of Sydney Sydney Australia

**Keywords:** eHealth, electronic health record, web-based intervention, implementation fidelity, user engagement, mixed methods, cardiovascular disease, primary health care, mobile phone

## Abstract

**Background:**

Growing evidence supports the benefits of eHealth interventions to increase patient engagement and improve outcomes for a range of conditions. However, ineffective program delivery and usage attrition limit exposure to these interventions and may reduce their effectiveness.

**Objective:**

This study aims to evaluate the delivery fidelity of an eHealth intervention, describe use patterns, compare outcomes between low and high users, and identify mediating factors on intervention delivery and receipt.

**Methods:**

This is a mixed methods study of an internet-based intervention being evaluated for effectiveness in a randomized controlled trial (RCT). The intervention comprised medication and cardiovascular disease (CVD) risk data uploaded from the primary care electronic health record (EHR); interactive, personalized CVD risk score estimation; goal setting and self-monitoring; an interactive social forum; and optional receipt of heart health messages. Fidelity was assessed over 12 months. Trial outcomes were compared between low and high users. Data sources included program delivery records, web log data, trial data, and thematic analysis of communication records.

**Results:**

Most participants in the intervention group (451/486, 93%) had an initial training session conducted by telephone (413/447, 92.4% of participants trained), with a mean duration of 44 minutes (range 10-90 minutes). Staff conducted 98.45% (1776/1804) of the expected follow-ups, mostly by telephone or email. Of the 451 participants who commenced log-ins, 46.8% (211) were categorized as low users (defined as at least one log-in in 3 or fewer months of follow-up), 40.4% (182) were categorized as high users (at least one log-in in more than 3 months of follow-up), and 12.8% (58) were nonadopters (no log-ins after their training session). The mean log-in frequency was 3-4 per month in ongoing users. There was no significant difference between the groups in the primary trial outcome of adherence to guideline-recommended medications (*P*=.44). In unadjusted analyses, high users had significantly greater eHealth literacy scores (*P*=.003) and were more likely to meet recommended weekly targets for fruit (*P*=.03) and fish (*P*=.004) servings; however, the adjusted findings were not significant. Interactive screen use was highest for goal tracking and lowest for the chat forum. Screens with EHR-derived data held only an early interest for most users. Fidelity measures (reach, content, dose delivered, and dose received) were influenced by the facilitation strategies used by staff, *invisible* qualities of staff-participant communication, and participants’ responsiveness to intervention attributes.

**Conclusions:**

A multifeature internet-based intervention was delivered with high fidelity to the RCT protocol and was regularly used by 40.4% (182/451) of users over 12 months. Higher log-in frequency as an indicator of greater intervention exposure was not associated with statistically significant improvements in eHealth literacy scores, lifestyle changes, or clinical outcomes. Attributes of the intervention and individualized support influenced initial and ongoing use.

## Introduction

### Background

Cardiovascular disease (CVD) accounts for approximately one-third of deaths globally [1], and effective prevention remains the cornerstone of efforts to reduce disease morbidity and mortality. Emphasis is on improving modifiable lifestyle factors, such as tobacco smoking, obesity, unhealthy diet, low physical activity, and alcohol consumption, and controlling high-risk conditions such as hyperglycemia, hypertension, and hyperlipidemia [2]. Technology-based approaches have the potential to support self-care, self-monitoring, and adoption of CVD prevention recommendations and more active interactions between patients and health care providers for managing chronic conditions [3-5]. Evidence for the effectiveness of internet-based strategies for CVD risk factor control is promising [6,7] but considered inconclusive [8].

Studies of internet-based interventions to support lifestyle behavior modification identify attrition of web use among the key reasons for suboptimal exposure or participation, which in turn influences uptake, adherence, and potential effectiveness [9-12]. The reasons are likely multifactorial: interventions targeting behavior change often consist of many interactive components and functions for voluntary use by recipients in home-based or nonclinical settings—attributes of intervention complexity [13]. Engagement with such interventions is characterized not only by subjective user qualities, such as interest, motivation, and sensory and intellectual satisfaction [14], but also by how these intersect with website use or behavior [15]. However, self-reported use of internet-based health resources generally does not objectively describe actual website interactions over time. This presents a gap in the fuller understanding of the activities undertaken at such websites [16]. Information about user activities has important implications for design and functionality decisions that can influence initial adoption and ongoing use [17].

Within a process evaluation, it is therefore useful to examine the fidelity of intervention delivery and receipt in terms of actual use behavior and a fuller understanding of the content that most influences user interaction. Intervention fidelity encapsulates both the implementation and delivery by providers and the treatment receipt and interaction with the intervention by the intended target audience [18]. Both dimensions can influence future program adaptation and scale-up [19]. Other proposed benefits of measuring or evaluating fidelity include ensuring internal program validity [20] and identifying which intervention elements affected participant responsiveness or reaction [21]. It is further recommended that these analyses precede trial outcome assessments to avoid bias in their interpretation [13,22].

### Objectives

Using data from a cohort of participants at moderate to high risk for CVD within a randomized controlled trial (RCT), this study aims to (1) evaluate the delivery fidelity of a consumer-focused web application with integration of data from primary health care electronic health records (EHRs), (2) describe objectively measured patterns of website usage and compare RCT outcomes between low and high users, and (3) identify mediating factors on delivery and receipt of the intervention that may explain the engagement and interaction patterns observed.

## Methods

### Design

A mixed methods study was nested within a process evaluation of the Consumer Navigation of Electronic Cardiovascular Tools (CONNECT) RCT (Australian New Zealand Clinical Trials Registry ID: 12613000715774). Protocols detailing the RCT and process evaluation have been previously reported [23,24]. The process evaluation is multifactorial. The focus of this study was delivery fidelity; other aspects of the overall evaluation that have been previously reported [25,26] are not part of this study. Ethics approval was obtained from the University of Sydney (reference 2013/716) and the New South Wales Aboriginal Health and Medical Research Council (reference 959/13).

### Participants

Adults with or at increased risk for CVD who had access to an internet-enabled device at least once per month and who could provide written informed consent were eligible to participate in the RCT. It was not a condition of enrollment that participants met any specific threshold of digital literacy or skill. Interest and willingness to use their device for study participation were required, but the extent of dependency on others to use their device for study purposes was not formally assessed at enrollment. Therefore, the digital support needs of individual participants were initially unknown to the staff who delivered the intervention. eHealth literacy scores were among the baseline data obtained from all those recruited but were not analyzed until completion of the RCT. Recruitment was conducted from 24 primary health care services in Sydney, Australia. Of the 934 participants who enrolled, 52.0% (486) were allocated to the intervention group and 48.0% (448) were allocated to the control group. The control group received usual care from their primary health care provider; the intervention group received their usual care plus access to the eHealth intervention. This evaluation addresses the fidelity of intervention delivery in the latter group of participants.

### Trial Outcomes

Briefly, the RCT tested the effectiveness of an eHealth intervention integrated with the primary care EHR for improving CVD risk factor control through better adherence to prescribed medications and lifestyle recommendations. The main trial results have been published elsewhere [27]. In summary, there was no significant difference between the control and intervention groups in the primary outcome of adherence to guideline-recommended medications, as defined by the proportion of days covered by guideline-recommended medications using pharmacy dispensing data (29.9% vs 32.8%; *P*=.48). There was little heterogeneity in the outcomes observed for prespecified subgroups, such as those with and without established CVD. However, the intervention was associated with improvements in 2 secondary outcomes: higher eHealth literacy scores (*P*<.001) and increased self-reported physical activity (87.0% vs 79.7%; *P*=.02).

### Intervention

Participants in the intervention group received access to a purpose-built, multi-feature web application securely linked to data within their primary care EHR. The extensive, systematic process by which the application was co-designed and evaluated for usability in patients with CVD is detailed elsewhere [28]. The logic model linking program inputs to expected user uptake activities within the CONNECT RCT has been described previously [24]. Briefly, software to enable EHR data transfer (RecordConnect, Extensia Pty Ltd) was installed into the clinical software systems of participating primary care providers. After each medical encounter, changes or additions made within selected data fields of the EHR were uploaded to the consumer-facing website by the provider. Concurrently, participants could enter and track measurements in charts displaying trends, targets, and current results for personal biometric and pathology data related to vascular risk factors. Other personalized interactive features included absolute CVD risk score and heart age estimation, goal setting and tracking for healthy eating, physical activity, smoking cessation and emotional well-being, and updateable display of diagnoses and prescription medications with accompanying consumer information resources from national peak health bodies. Participants could also read and contribute to a closed chat forum page and could opt to receive semipersonalized heart health messages via email and/or text message format. Participants could receive an alert when their portal was updated from the EHR and could select their preferences for the heart health messages content from topics such as healthy lifestyle, goal reminders, and medication knowledge. The text message content was adapted from messages that our colleagues initially developed and tested for use with patients with CVD [29]. In this study of patients with similar demography and diagnoses to the earlier study, the multidisciplinary research team further reviewed and expanded the messages to ensure alignment with current Australian guidelines for primary and secondary prevention of CVD. The intervention was intended to take place in real life; it was home-based and without mandatory task completion, data entry, or counselor supervision. As a self-directed intervention, the frequency and extent of use were entirely controlled by the participants, although the study staff encouraged them to interact with and revisit the information and features.

### Staff Support

Project staff consisting of registered nurses, dietitians, and pharmacists provided support for patients throughout the intervention period. Although these staff encouraged participants to interact with and revisit the information and features, they were not expected to formally counsel participants about risk factor management pertaining to their health circumstances.

During the initial one-to-one telephone-based or face-to-face training session, study staff provided participants with their *go-live* log-in information and orientation to the website features and navigation, and they answered initial technical and clinical questions. In terms of the quality of the intervention presented to participants, portals were checked before each participant’s go-live session to ensure that data uploads from the EHR to the portal were current and that any identified screen errors were rectified before go-live. Thereafter, follow-up contact occurred at 4 scheduled time points (weeks 2, 6, 12, and 26) to answer questions and encourage website interaction and return visits. All scheduled and ad hoc contacts were documented on a standard form and included comments made by the participants and/or notes made by the staff. Participants who did not perform the initial training session with staff were sent self-directed materials; if they initiated log-ins, staff then made the required scheduled contacts.

Scheduled follow-up contact was by telephone unless the participant indicated a preference for email communication. Wherever possible, the staff member who conducted the go-live training conducted the scheduled follow-up with the participant and responded to their email and telephone communication. At each follow-up time point, the staff made at least two attempts to contact participants by telephone; if unsuccessful, an email was sent. To conduct the training sessions and scheduled follow-ups, standard operating procedures were developed, refined, and adopted throughout the RCT to optimize implementation consistency. A separate group of staff, blinded to the intervention allocation, conducted study outcome assessments and did not participate in the delivery of the intervention.

### Data Sources

A total of five core measures of intervention delivery fidelity derived from concepts in digital health engagement [15] and guidance on process evaluation and implementation research, [30] were evaluated before knowledge of the RCT outcomes: reach, content fidelity, dose delivered, frequency and duration, and exposure (Table 1).

**Table 1:** Fidelity measures.

Fidelity measure	Description [15,30]	Data sources
Reach	Proportion of the intended target audience that participates in all or part of the intervention	Program delivery records
Content fidelity	The intervention content is delivered in the intended manner and quality	Program delivery records
Dose delivered	The amount of the intervention components that were provided to participants	Program delivery records
Frequency and duration	How long the intervention was implemented as intended in the trial design and how often participants made contact with the intervention	Program delivery records
Dose received or exposure	How much of the activities or components was read, viewed, or used for the intended duration	Web log files

Program delivery records regarding go-live training, and scheduled and ad hoc communication with participants, were reviewed. Participant factors affecting intervention delivery and exposure were gathered from textual data within the email and telephone records. These 2 fidelity measures closely relate to the 2 stages of a program’s logic model: the intervention inputs and the intervention uptake [20].

Website use was central to the *dose* of intervention the participants received. Web log data were used to quantify use in terms of frequency and intensity over the 12 months of study follow-up. Go-live and subsequent session or log-in numbers and screens viewed per month were available for each participant. Session or page view duration and the number of daily interactions were not available. To further characterize exposure, participants were categorized as nonadopters, low (lower) users, or high (higher) users. The number of months in which a participant logged into the application at least once was counted during the 12-month follow-up period to categorize use. Nonadopters were defined as those who logged into the application only once. Low users were defined as those who logged into the application at least once per month in 3 or fewer months (eg, 12 log-ins in month 4 and 5 log-ins in month 7 of follow-up would be categorized as low use). High users were defined as those who logged into the application at least once per month in more than 3 months (eg, 12 log-ins in month 4, 5 log-ins in month 7, 3 log-ins in month 8, and 1 log-in in month 11 of follow-up would be categorized as high use). Hence, the definitions prioritized returning or ongoing log-in activity, often in nonconsecutive months, over the absolute number of log-ins. The log-in frequency data were skewed. Three months was chosen as the cutoff based on the median number of monthly log-ins over 12 months of follow-up. In the absence of an agreed standard for low versus high use of such interventions, the criteria used in this study resulted from an exploratory analysis of the log-in data at the completion of the RCT rather than from definitions set a priori. Optional email and/or SMS text messaging receipt was compared with website use to quantify the combination of passive and active content exposure.

### Data Analysis

Descriptive statistics were used to describe and compare the format, the time required, and the content of all staff-participant contact episodes during the go-live and study follow-up periods, thus indicating resource needs for implementing the intervention content as planned. Textual data in the participant contact records were coded for similarities in ideas, and themes were identified inductively to describe the broader issues surrounding implementation needs and participant responsiveness [31]. The themes identified as influencing delivery and uptake were then merged with the original program logic model to indicate where they affect assumptions about program function and could be important implementation considerations for future similar interventions.

The demographic and clinical characteristics of the 3 user subgroups (nonadopters, low users, and high users) were compared using baseline data from the RCT database. The use patterns and characteristics of different types of user groups may be important for the future design of portal functions to optimize adoption. Subanalyses of RCT outcomes were also performed according to the 3 subgroups. For the subanalyses, the nonadopters and low users were combined into 1 group because of the small numbers in the former group. For the primary outcome subanalysis, adherence to guideline-recommended medication was calculated using the proportion of days covered from national pharmaceutical dispensing data. If a participant had a proportion of days covered of ≥80%, the participant was considered adherent to the guideline-recommended medication. Adherence (yes or no) was analyzed using a logistic regression model with treatment as an independent variable. Relative risk with 95% CIs was estimated by comparing high and low users. The primary subanalysis was adjusted for age, sex, and diabetes status.

In the secondary and tertiary outcome subanalyses, for categorical variables where a difference in proportions between high and low users at 12 months was calculated, a chi-square test of independence was used. If cell counts were too small, Fisher exact test was used. For continuous variables where a difference in mean value between high and low users at 12 months was calculated, an independent samples *t* test was used. Statistical significance was set at *P*<.05. The tertiary outcome subanalysis was adjusted for baseline values and analyzed using the ANCOVA (analysis of covariance) model. Analyses were conducted using Excel (Microsoft Office Professional Plus 2016) and SAS version 9.4.

## Results

### Intervention Reach

**Figure 1:**
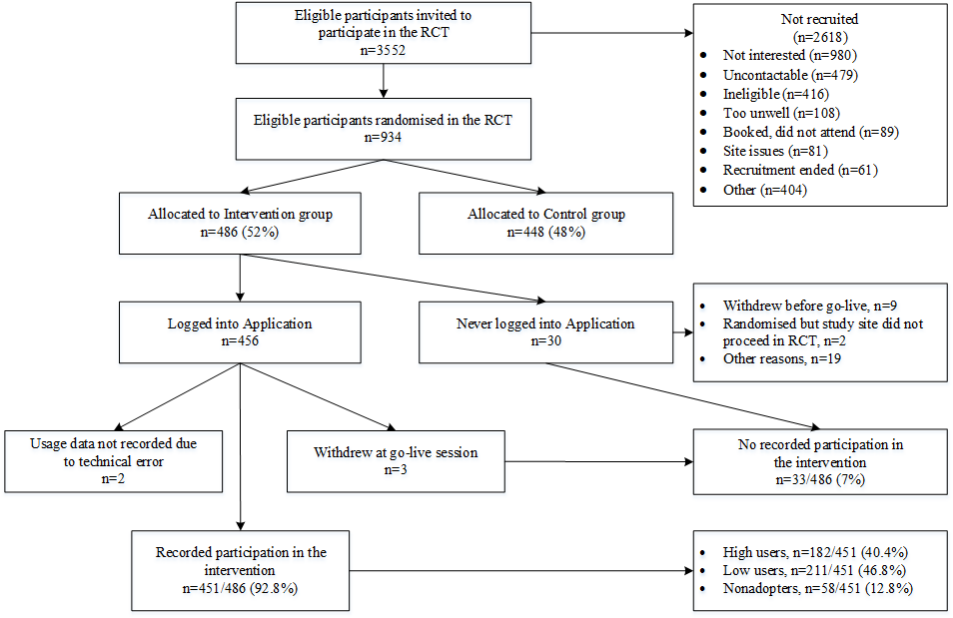
Intervention reach. RCT: randomized controlled trial.

Between November 2014 and May 2017, 934 eligible patients were enrolled in the RCT. Of these, 486 (52.0%) were allocated to the intervention group. The flow of the participants in the intervention group is shown in Figure 1. Participants exposed to all or part of the intervention (451/486, 92.8%) were defined as being registered in the shared record software (RecordPoint) *and* logged into the app *and* used at least one feature or component at least once. Reasons for part-participation were (1) participants opting out of system-generated SMS text messaging or email content (although they could still access the website or rejoin system-generated content at any time), (2) participants stopping log-ins to the website but remaining opted in to system-generated SMS text messaging and/or email content, and (3) participants withdrawing from the study after go-live but before completion of the follow-up period.

### Intervention Content Delivery

Of the 486 participants allocated to the RCT intervention group, 99.6% (484/486) had a personalized, secure website populated with data from their EHRs. A study site that discontinued participation left 2 enrolled participants without access to the intended intervention.

In total, 2.7% (13/486) of the cohort withdrew at or before go-live training, leaving 97.3% (473/486) to undergo training. Of these, 94.5% (447/473) received the training session with staff as intended, and 6% (26/473) who could not keep either their telephone or their in-person appointment were mailed the self-directed materials, enabling them to log in and orient themselves to the application and receive staff assistance if they chose to do so. Of the 451 participants with a recorded go-live, most (447, 99.1%) received data uploads from their EHR for a full follow-up period of 12 months. Four participants from a primary health care site that closed within the course of the study did not have EHR-sourced data updated in their portal for their full follow-up period. Most log-in sessions by participants occurred on laptops, desktop computers, and tablets, and a minority of the log-in sessions occurred on smartphones.

An automated system sent messages at a preset, tapering frequency, commencing at the go-live session. Each month, 20% of the message delivery records were randomly selected and reviewed for compliance with the intended delivery schedule. Across both formats, delivery accuracy ranged from 86% to 100%. A severe disruption to weekly volumes of SMS text message delivery was identified by staff in March 2017. Program records indicated the text messages that were queued to be sent but did not indicate the actual message receipt; hence, nonreceipt would go unnoticed unless participants reported the problem. Once notified, programmers traced and rectified the source of the problem within the notification system; however, in the interim, a small number of participants did not receive the heart health content delivered via SMS text messaging.

### Scheduled and Ad Hoc Participant Contact

Intervention training occurred over the telephone (413/447, 92.4%) or in-person (34/447, 7.6%). The mean training time for each format was 44 minutes (range 17-120 minutes) and 38 minutes (range 10-90 minutes), respectively. The characteristics of the scheduled participant support after training are summarized in Table 2.

Overall, 1804 follow-up calls were predicted based on 4 calls to each of the 451 participants, and 98.45% (1776/1804) were achieved. Of those, 80.29% (1426/1776) were verbal conversations and 19.71% (350/1776) were communications via email or voicemail messages. No contact was attempted with participants who, in the time since the previous scheduled contact, requested no further calls from the study staff. Overall, the median time required for follow-up communication was 5 minutes. Technical problems or *how to* questions comprised most of the reported problems, and although they reduced over the 4 contact time points, forgotten passwords and SMS text messaging or email faults or preferences together accounted for approximately half of the technical assistance at week 26.

Unlike the scheduled contact, most of the ad hoc contact was initiated by participants (286/483 episodes, 59.2%) and involved 285 unique participants, representing 63.2% (285/451) of the participants. Ad hoc contact was more frequent by email (363/557, 65.2%) than by telephone (158/557, 28.4%) and required an average of 8.5 (SD 7.5) minutes of staff time (range 1-80 minutes; median 5 minutes). Most of the contact was for resolution of technical problems such as log-in or password problems (244/483, 50.5%), turning message receipt on or off (66/244, 27.0%), or discussing tracking or self-monitoring measurements (53/244, 21.7%). General feedback and miscellaneous administrative issues accounted for 26.3% (127/483). Content data were incomplete in 23.2% (112/483) of the contact logs.

**Table 2:** Characteristics of scheduled intervention participant support during study follow-up.

Characteristic				Scheduled follow-up after go-live training session								
				Week 2		Week 6		Week 12		Week 26		All weeks
Contact episodes initiated by project staff (n)				451		446		442		437		1776
**Communication format (where specified)**												
	**Contact attempts, n (%)**			458 (25.46)		450 (25.01)		446 (24.79)		445 (24.74)		1799 (100.00)
		Telephone	432 (94.32)		427 (94.89)		425 (95.29)		423 (95.06)		1707 (94.88)	
		Email	20 (4.37)		21 (4.67)		20 (4.48)		19 (4.27)		80 (4.45)	
		SMS	5 (1.09)		2 (0.44)		1 (0.22)		3 (0.67)		11 (0.61)	
**Time taken (min)**												
	Total			2767		2606		2564		2576		10,513
	Mean (SD)			6 (4.8)		6 (4.1)		6 (4.6)		6 (3.8)		6 (4.3)
	Minimum, maximum			1, 45		1, 45		1, 60		1, 35		1, 60
	Median (Q1^a^; Q3^b^)			5 (5, 5)		5 (4, 5)		5 (5, 5)		5 (5, 5)		5 (5, 5)
**Problem category (where specified), n (%)**												
	No problems reported by participant^c^			320 (83.55)		332 (90.22)		309 (90.88)		297 (88.66)		1258 (88.22)
	More training (technical steps and/or explaining clinical information)^d^			9 (1.99)		4 (0.90)		4 (0.90)		4 (0.92)		21 (1.18)
	**Technical problem or *how to* inquiry^c^**			57 (12.64)		33 (7.40)		30 (6.79)		23 (5.26)		143 (8.05)
		Log-in problem or password reset	18 (31.58)		7 (21.21)		13 (43.33)		12 (52.17)		50 (34.97)	
		Turning automated heart health message tip receipt on or off; faulty email delivery	19 (33.33)		11 (33.33)		7 (23.33)		11 (47.83)		48 (33.57)	
		Tracking or self-monitoring measurements	12 (21.05)		10 (30.30)		6 (20.00)		2 (8.70)		30 (20.98)	
	Request to withdraw or change study participation status			3 (0.67)		2 (0.45)		2 (0.45)		1 (0.23)		8 (0.45)

^a^Q1: first quartile.

^b^Q3: third quartile.

^c^The denominator includes telephone and email communication.

^d^The denominator is contact episodes in which staff directly spoke to the participant.

### Intervention Log-In Frequency and Duration

**Figure 2:**
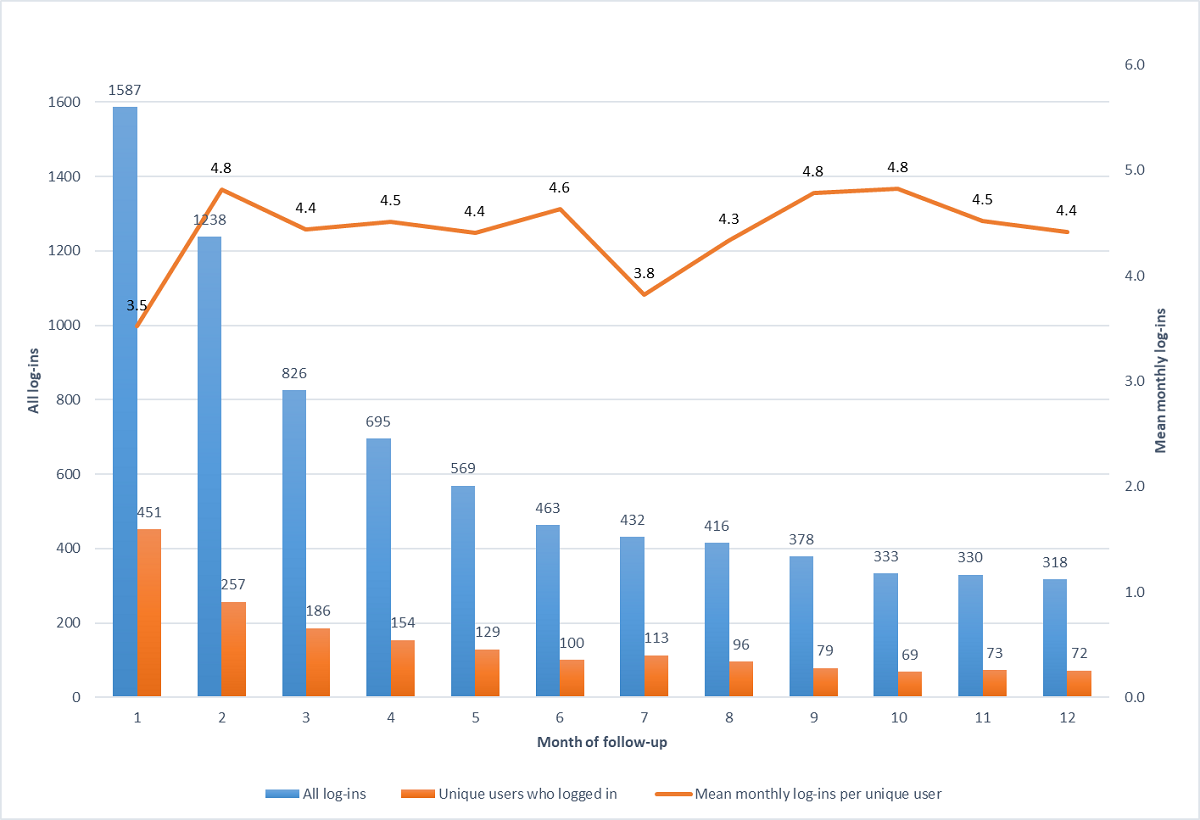
Characteristics of log-in activity.

The overall log-in activity pattern was used to indicate the frequency with which participants made contact with the intervention. The highest number of log-ins (n=1587) occurred in month 1 of participation, with subsequent monthly log-ins decreasing steeply by month 6 (n=463) and then remaining relatively stable from months 7 to 12 (Figure 2). Log-in activity by unique users began with all users (n=451) logging in at least to go-live. Unique user log-ins declined markedly in month 2 (257/451, 56.9%) and thereafter were made by up to 50.9% (200/393) of those who made any use of the intervention (after nonadopters were excluded). The mean log-in frequency among ongoing unique users was 3 to 4 times per month over 12 months. The median monthly log-in frequency was 4.5 (Q1, Q3: 4.4, 4.7) among ongoing users.

### Intervention Exposure

**Figure 3:**
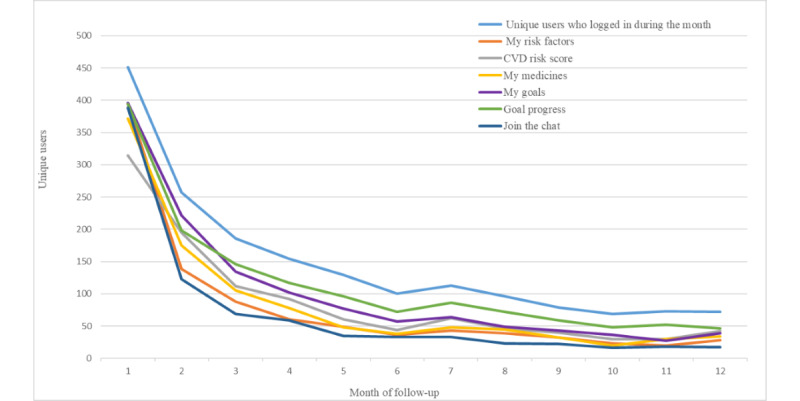
Number of unique users who log in and access interactive screens per month of follow-up. CVD: cardiovascular disease.

Excluding the nonadopters (58/451), monthly unique users dropped below 200 from month 3 among participants who continued to make any use of the intervention (393/451) and below 100 from month 8 (Figure 3). Only goals tracking was accessed by more than 50 unique users per month for 12 months. In terms of goal setting, 86.0% (388/451) of all users set goals for healthier lifestyle behavior, and most goals were for healthier eating and physical activity. More people set goals than returned to track their goal achievements. Overall, the social media or chat forum feature was the least subscribed, being visited by 12.7% (50/393) of unique users per month beyond month 4. Contributions to the forum by participants ranged from 0 to 12 postings each month.

**Figure 4:**
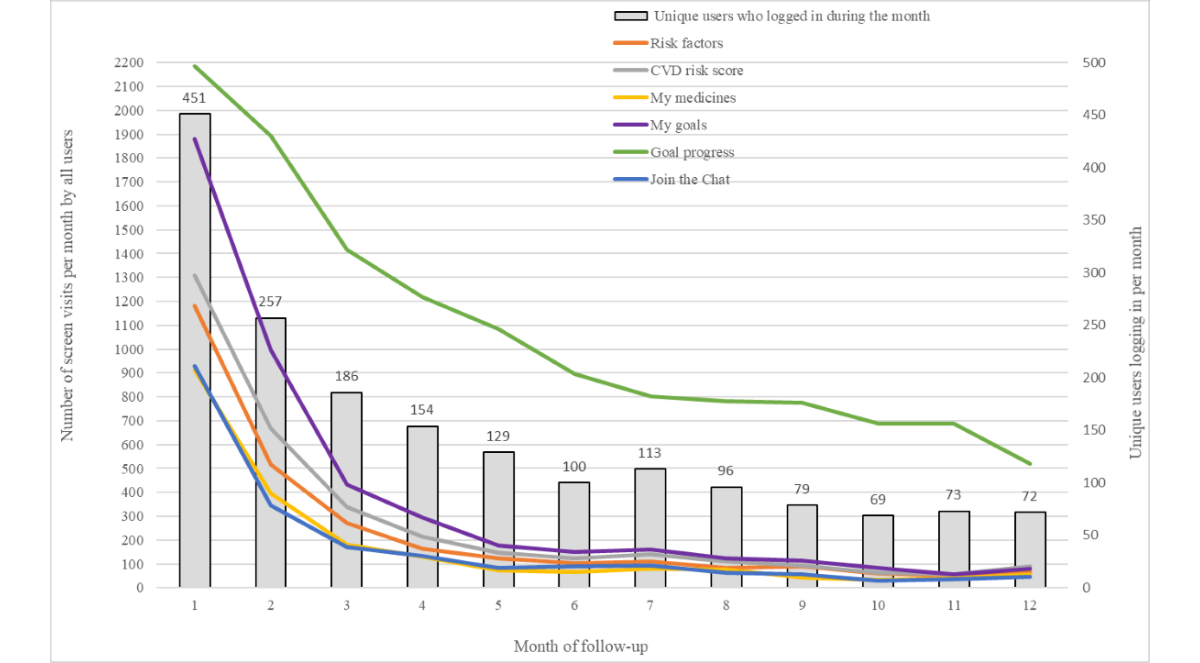
Intensity of screen visits over 12 months of follow-up. CVD: cardiovascular disease.

In terms of the intensity of screen visits over time by unique users, visits to track goal progress markedly exceeded visits to all other screens over each of the 12 months, even though fewer unique users were logging in (Figure 4). By month 12, for example, 18.3% (72/393) of unique users made approximately 500 visits to the goal-tracking screen. In comparison, in the same month, there were fewer than 100 visits to each of the other interactive features, suggesting that electronic goal tracking was a valued feature determining returning log-ins. Overall, personal goal setting, risk factor monitoring, and CVD risk score estimation were accessed more than the chat forum and medicine features, with the highest interest in the earlier months.

### Subanalysis of High Versus Low Intervention Users

#### Baseline Characteristics

Of the 451 participants with usage records, 12.8% (58/451) were nonadopters, 46.8% (211/451) were low users, and 40.4% (182/451) were high users (Table 3). There were no major baseline differences between these groups in terms of demographic and biometric data, CVD risk status, or other health conditions. The characteristics of all intervention group participants in this evaluation study mirrored those of the overall RCT cohort [27], although the proportion of Aboriginal and Torres Strait Islander Australians was higher than in the RCT (4.9% compared with 4.0%) and slightly higher than the proportion of Aboriginal and Torres Strait Islander representation in the general Australian population in the most recent census (3.3%) [32]. The mean difference in eHealth literacy scale (eHEALS) scores between low and high users was significant (1.13; 95% CI 0.53-1.72; *P*<.001). Most of the nonadopters (32/58, 55%) received school education only, whereas postschool education was more frequent in the low (150/211, 71.1%) and high (142/180, 78.9%) users.

**Table 3:** Baseline characteristics by intervention user subgroups.

Characteristics			Nonadopters^a^ (n=58)	Low users^b^ (n=211)	High users^c^ (n=182)	Total (N=451)
Age (years), mean (SD)			66.8 (8.2)	66.8 (8.2)	67.2 (8.7)	67.0 (8.4)
Male, n (%)			40 (68.9)	158 (74.9)	147 (80.8)	345 (76.5)
**CVD^d^ risk, n (%)**						
	Existing		20 (34.5)	91 (43.1)	77 (42.3)	188 (41.7)
	High		38 (65.5)	120 (56.9)	105 (57.7)	263 (58.3)
**Ethnicity, n (%)**						
	Indigenous Australian		6 (10.3)	10 (4.7)	6 (3.3)	22 (4.9)
	White		46 (79.3)	176 (83.4)	161 (88.5)	383 (84.9)
	South Asian		3 (5.2)	3 (1.4)	6 (3.3)	12 (2.7)
	Other Asian		1 (1.7)	3 (1.4)	3 (1.6)	7 (1.6)
	Other		2 (3.4)	19 (9.0)	6 (3.3)	27 (5.9)
**Education level, n (%)**						
	Secondary school or below		32 (55.2)	61 (28.9)	38 (21.1)	131 (29.2)
	Technical or vocational qualification, or above		26 (44.8)	150 (71.1)	142 (78.9)	318 (70.8)
**Annual household income, n (%)**						
	<Aus $104,000 (US $79,366)		34 (58.6)	120 (56.9)	114 (63.0)	268 (59.6)
	≥Aus $104,000 (US $79,366)		24 (41.4)	91 (43.1)	67 (37.0)	182 (40.4)
**CVD risk factors**						
	BMI (kg/m^2^), mean (SD)		29.4 (6.74)	30.3 (5.45)	29.5 (5.22)	29.8 (5.54)
	BMI ≥30 kg/m^2^, n (%)		22 (37.9)	101 (47.9)	69 (37.9)	192 (42.6)
	Waist circumference (cm), mean (SD)		104.5 (18.1)	107.0 (15.4)	104.9 (13.1)	105.8 (14.9)
	SBP^e^ (mm Hg), mean (SD)		138.7 (16.7)	136.4 (15.6)	139.1 (16.1)	137.8 (15.9)
	DBP^f^ (mm Hg), mean (SD)		79.7 (11.4)	78.4 (10.6)	79.4 (10.5)	79.0 (10.7)
	Current smoker^g^, n (%)		10 (17.2)	22 (10.6)	18 (9.9)	50 (11.2)
	LDL^h^ cholesterol (mmol/L), mean (SD)		2.6 (1.09)	2.6 (1.09)	2.6 (1.0)	2.6 (1.05)
	HDL^i^ cholesterol (mmol/L), mean (SD)		1.4 (0.49)	1.3 (0.37)	1.3 (0.36)	1.3 (0.39)
	**HbA_1c_^j^**					
		Participant, n (%)	21 (36.2)	71(33.6)	49(26.9)	141(31.3)
		Mean (SD)	6.8 (1.0)	7.0 (1.2)	6.9 (1.4)	6.9 (1.3)
**Comorbidities, n (%)**						
	Previous stroke		6 (10.3)	20 (9.5)	14 (7.7)	40 (8.9)
	Coronary heart disease		15 (25.9)	78 (36.9)	65 (35.7)	158 (35.0)
	Atrial fibrillation		6 (10.3)	21 (9.9)	17 (9.3)	44 (9.8)
	Diabetes mellitus		22 (37.9)	75 (35.5)	50 (27.5)	147 (32.6)
	COPD^k^ or emphysema		4 (6.9)	17 (8.1)	9 (4.9)	30 (6.7)
**Self-reported medication use, n (%)**						
	Antihypertensive		36 (72)	128 (61.8)	106 (60.6)	270 (62.5)
	Lipid-lowering		26 (52)	123 (59.4)	94 (53.7)	243 (56.3)
	Antithrombotic		16 (32)	86 (41.5)	71 (40.6)	173 (40.0)
**PBS^l^ medication use, n (%)**						
	Antihypertensive^m^		31 (62)	106 (51.2)	89 (50.9)	226 (52.3)
	Lipid-lowering^m^		18 (36)	79 (34.8)	72 (41.1)	169 (39.1)
	Antithrombotic^m^		3 (6)	38 (18.4)	17 (9.7)	58 (13.4)
eHEALS^n^ mean (SD)			24.6 (7.7)	26.7 (6.3)	28.5 (5.7)	27.1 (6.4)

^a^Nonadopter is defined as a participant who logged into the app only once in total.

^b^Low user is defined as a participant who logged into the app at least once in 3 or fewer months of follow-up.

^c^High user is defined as a participant who logged into the app at least once in more than 3 months of follow-up.

^d^CVD: cardiovascular disease.

^e^SBP: systolic blood pressure.

^f^DBP: diastolic blood pressure.

^g^Eight participants with missing carbon monoxide breath test results.

^h^LDL: low-density lipoprotein.

^i^HDL: high-density lipoprotein.

^j^HbA_1c_: hemoglobin A_1c_.

^k^COPD: chronic obstructive pulmonary disease.

^l^PBS: pharmaceutical benefits scheme.

^m^Forty-three participants withdrew consent for the use of their pharmaceutical benefits scheme data.

^n^eHEALS: eHealth literacy scale. Maximum score is 40.

#### RCT Outcomes

There was no significant difference in the primary outcome of adherence to guideline-recommended medication between the low- and high-user groups (*P*=.44), although the proportion of participants that was adherent increased by 5.7% in the high-user group but only by 3.1% in the low-user group. In the unadjusted analyses, compared with the low-user group, the high-user group had significantly higher eHEALS scores and mean numbers of fruit and fish serves per week; however, these differences were not significant after adjustment for baseline scores. Compared with low users, high users also had nonsignificant higher adherence rates to blood pressure–lowering therapy and statin medications, meeting Australian guideline targets for both blood pressure and low-density lipoprotein cholesterol, and doing at least one thing to control their salt intake.

#### Exposure to Email and SMS Content

Most participants in each of the subgroups opted to receive messages for more than 3 months of follow-up: 96.2% (175/182) of high users, 86.9% (166/191) of low users (data missing for 20/211, 9.5% of low users), and 90% (52/58) of nonadopters.

### Factors Mediating Intervention Delivery and Receipt or Early Adoption

**Figure 5:**
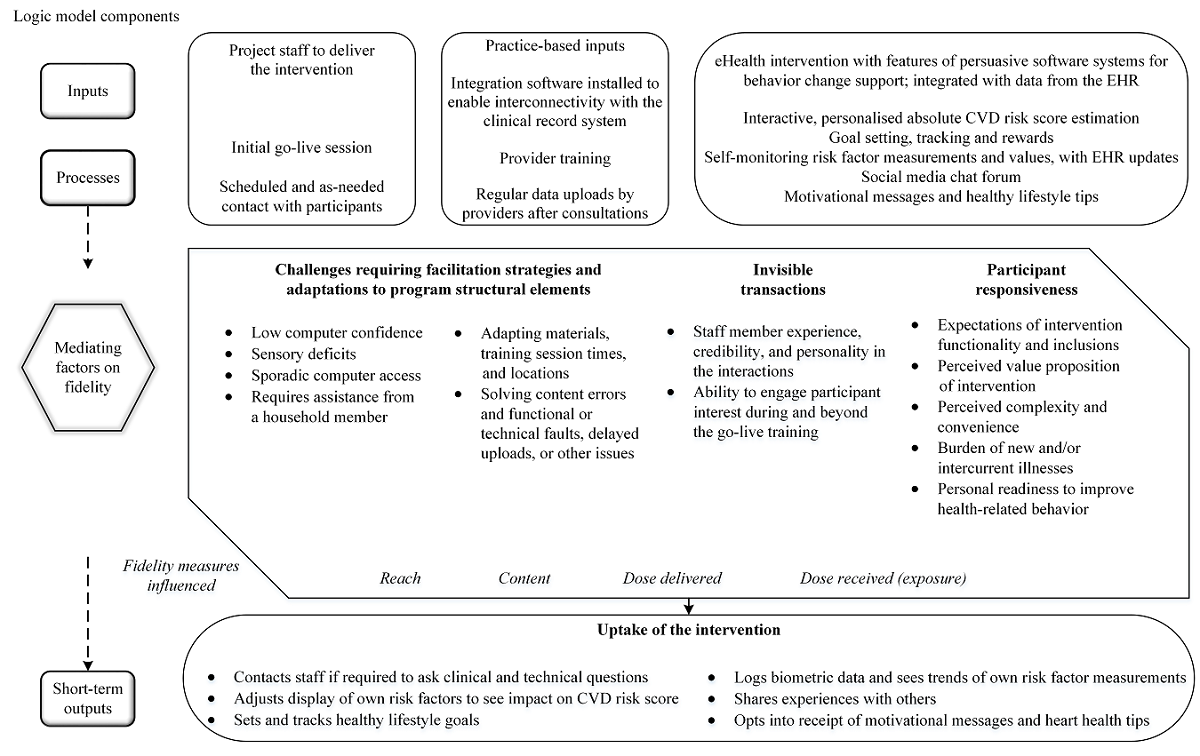
Modified logic model showing mediating factors on the relationship between the intervention processes and outputs. CVD: cardiovascular disease; EHR: electronic health record.

At the completion of the RCT, 3 factors were identified from the review of program delivery and participant communication records as having influenced intervention dose delivery and receipt, in relation to the stages of the program logic model (Figure 5).

#### Facilitation Strategies by Staff and Adaptation of Structural Elements of Program Delivery

Standardized intervention delivery was subject to some variation and adaptation, as different participant needs were identified by staff conducting the go-live training and follow-up communication. After reviewing the communication records with participants, a range of facilitating strategies emerged as having been important to intervention receipt; they are summarized in Textbox 1. The conduct of go-live training was always adapted to enable those with hearing or speech impairments, or less computer confidence, to take part. A paper-based reference guide was provided to those who found website navigation and functions difficult to recall. Telephone-based training with participants with low skills or memory impairment extended the time requirements and underscored the need for clear, simple instructions with less technical jargon; setting up website shortcuts and browser bookmarks to simplify return log-ins; allowing time to practice on interactive screens; and split-session training. Some participants needed a family or household member to be present or required their training appointment outside business hours.

**Facilitation strategies required to support intervention delivery and receipt.**

Facilitating strategies identified from communication records:

- Face-to-face go-live training offered to those unwilling to do so by telephone
- Go-live scheduling outside business hours (eg, evenings or Saturday mornings)
- Inclusion of a carer or family member in go-live training or ongoing communication to facilitate use of the intervention
- A self-directed paper guide for participants who
  required a nonelectronic reference to improve confidence with navigation of the website and its features
  had hearing or speech impairments that would make telephone-based go-live training unsuitable
  did not participate in go-live training with staff
- Retraining of website navigation skills at any time in the follow-up period
- Resolution of technical questions that could reasonably be done by the participant without staff assistance (eg, changing preferences or settings within the app)
- Trouble-shooting delayed upload of recent measurements or pathology test results from the electronic health record, which may have revealed a fault with the data integration software at the health service
- Sending a courtesy email to a participant when an error or technical problem within their application had been corrected or if a required correction was delayed
- Removing or correcting erroneous biometric data entry within screens or charts that were not editable by the participant

#### Invisible Transactions

Staff member experience and personality as a mediating factor on the quality of professional communication and interaction with participants and the ability to engage their interest during and beyond *go-live* training were not formally measured. It is uncertain if, and how much, the staff members’ credibility, trustworthiness, skill, and friendliness affected participants’ initial and ongoing willingness to engage with the intervention. Regardless, program delivery and communication records revealed that these qualitative influences were likely at play, although they could not be quantified. Staff notes about personal health topics raised by participants in calls and emails and several elements of the study design underscored the value of staff attributes, namely, (1) the personalized nature of EHR-derived data that often required clear, accurate explanations by telephone or email, without complex medical or technical jargon; (2) the remote intervention delivery and support arrangements that increased anonymity between parties and prevented face-to-face communication cues; and (3) the wide variation in participant ages, education, and digital literacy that necessitated more supportive approaches for some. During a call at week 26, a participant stated that she always felt more energized after calls with staff, suggesting that human contact may still be valued even after 6 months using a self-directed resource.

#### Participant Responsiveness

Four concepts appeared to influence participant responsiveness to the intervention, and hence log-in frequency and exposure, given that all components and content were available.

First, participants’ preferences for intervention functionality and inclusion influenced their reactions. The concept and presentation were appealing, but some participants’ expectations were not met. Comments included:

...I would like to see log space for blood sugar levels measured at home.

Go-live phone call

Simply adding data and reading all the information is not enough

Week 2 phone call

...I wish I had this when I had my heart attack. I am doing more walking and eating more vegetables than I ever have before. It keeps me on track all the time.

Week 6 phone call

A second and related expectation was the perceived value offered by the intervention. Some viewed it as minimally useful compared with their existing resources or habits for managing their health, suggesting the need for strong personal relevance of content or functions. Others gained motivation for their healthier lifestyle efforts and were prompted to log in. The descriptive comments included the following:

...After my angioplasty...the information was largely what had been covered in the hospital rehab program and did not really provide any additional motivation to me.

Email from a participant

...It’s been a big help...tracking goals has become my routine now. Email and SMS tips are reinforcing, it makes me want to go back into my app to update the tracker.

Week 26 phone call

Setting goals gave me an incentive to log on.

Email from a participant

Interestingly, the EHR data integration was of less value to participants who felt a close rapport with their care provider or for whom new data uploads would be infrequent:

...The tie-in with the [doctor] was good but not useful for me because I don’t go that often.

Week 26 phone call

...A lot of the Program I found was not useful to a person like me who has a good relationship with their GP and practice.

Email from a participant

A third issue affecting log-ins was perceived convenience compared with other digital health apps or devices. The intervention was useful for monitoring and assessing risk but not for fitness tracking, for example, which was a priority for some users. For example, participants described the concurrent use of commercial phone-based apps or a wearable device on which they could readily track their physical activity and dietary behaviors. Others were satisfied with the content from the automated messages and, therefore, logged in less often:

...I haven’t logged onto the app very often; however, I like receiving the tips, they remind me to be good.

Week 12 phone call

I use other apps to count calories and steps.

Week 26 phone call

...I am not much on the app; I prefer to access an app quickly on the phone with few clicks to see the essential info.

Week 6 phone call

Fourth, timing relative to other personal priorities affected intervention exposure. Participants described infrequent log-ins because they (or their spouse, for example) had a new or existing intercurrent illness with immediate priority. Examples included new diagnoses of cancer or other long-term conditions, unforeseen surgery, the demands of a new treatment or therapy, and frequent clinic appointments.

These responsibilities lowered engagement with the intervention. For others, study enrollment coincided with their readiness to improve their health-related behavior. A participant commented that his participation was “a wake-up call” that helped him to succeed with weight loss goals. Another stated after 6 months that “This is the catalyst that made me get stuck in.” Thus, the timing of participation was both a constraint and an enabler of intervention exposure.

## Discussion

### Principal Findings

This mixed methods evaluation examined the intervention delivery fidelity of a consumer-focused web application with data integration from the primary health care EHR and optional health message receipt. No single or uniform measure defines or quantifies fidelity [20,33], but core evaluation metrics of reach, content fidelity, and dose delivered [19,30] overall were fulfilled as intended. Adaptations to routine implementation, known as structural adaptations [34], were made to overcome individual barriers to intervention reach and receipt. Initial high-user log-ins dropped early in the follow-up period, a trend noted across such interventions more generally [35,36], and the unique user log-in rate tapered to a mean frequency of 3 to 4 per month. Progress tracking had the highest screen visit intensity for the longest duration. Screens with EHR-derived data generally held stronger interest in the early follow-up period. Email and SMS text message receipt augmented active participation by high users and strengthened content exposure in low users and nonadopters. Intervention nonadopters, low users, and high users were similar across a wide range of characteristics, suggesting that the website was amenable to use in general but not which characteristics were important for log-in frequency. The association of patient-level factors and log-in behavior would thus be of further research interest for improving design or excluding content [37]. More frequent intervention use was associated with nonsignificant differences in clinical measures and health-related lifestyle behaviors after 12 months. It is possible that the study was insufficiently powered to fully elucidate the impact of higher intervention exposure; however, in any event, the differences were small in absolute effect size and may not be clinically important. Further research could ascertain if the higher eHEALS scores noted at baseline in the high-user group are an important precursor to using digital health interventions and if more frequent use raises self-reported eHealth skills. Prescribed log-in activity in relation to the adoption of desired offline behaviors may be an area of further inquiry, particularly as website engagement has been shown to benefit physical activity and eating behaviors, even when not significantly associated with biological outcomes [38]. Qualitative data revealed important influencing factors on intervention delivery and receipt, a noted advantage of mixed methods inquiry in process evaluations [13], and a strength of this study. The original program logic model was used as not only a representation of the causal assumptions within the intervention [13] but also as a scaffold on which to show where the identified factors impacted intervention delivery and uptake [20].

Engagement with technology has been defined as a 4-stage cyclical process consisting of an initial point of engagement, the period of engagement, disengagement, and reengagement, with the stages having both shared and exclusive sensory-emotional and spatiotemporal attributes [39]. For example, the system’s novelty and esthetic attributes, combined with user emotions of motivation and interest, are especially important to initiation; attributes such as customization, feedback, and control that promote positive affect appear important to ongoing and return visits; and low-level interaction, boredom, or other negative emotions influence disengagement [39]. A recent systematic review found that changes to health status caused disruption or drop out, as did user perception of the technology’s compatibility with their routine, their own digital literacy, and relevance to their symptoms or (dis)abilities [40]. In the diffusion of innovations theory, individuals look to reduce their uncertainty about the consequences of adopting an innovation and to perceive a relative advantage if they do so [41]. Factors such as personal convenience, satisfaction, and suitability for needs are proposed to matter more than the intervention’s objective advantage. Notably, for this study, reliance on what the participants themselves chose to undertake was an important contextual factor for adoption [42], underscoring the significance of user-perceived relevance and value.

A more recent framework calls the desirability of the technology to the user its value proposition, and more complex interventions may be less likely to reach this threshold for adoption [43]. Personal relevance, program expectations, current health behavior, convenience, and so on, as identified in this study, concur with engagement factors for digital health interventions previously identified, particularly the themes of personal agency and motivation, personal life and values, and perceived quality of the intervention [44]. Notably, in a multidomain model for engagement with web-based interventions [14], the authors point out the utility of these determinants in any framework of assumptions about intervention use. The relevant framework in this study was the program logic model, in which the determinants derived from the qualitative data analysis were merged to help explain program uptake and what the intervention delivery required in practice (Figure 5). The perception of advantage from using such interventions may have an upstream influence on recruitment to the RCT; of the eligible invited participants who were not recruited, 37.43% (980/2618) gave their reason as *not interested* (Figure 1). Although reasons for disinterest among the RCT nonparticipants were outside the scope of this evaluation, we recommend that further studies be conducted to explore and define a value proposition for similar interventions targeting the primary health care context. Furthermore, the overall generalizability of findings may be improved with greater participation of primary care attendees in studies of digital interventions. The number of participants randomized represented 26.29% (934/3552) of those invited to participate in the RCT. Hence, there may be barriers and enablers of engagement and uptake that would be further understood with higher study participation in this setting.

Patient engagement with portals directly linked to an EHR has inconsistent definitions of both adoption [17] and active use, ranging from at least one use, [45] to more than 1 log-in every 4 months, [37] and at least two log-ins in 12 months [16]. Other studies of web-based lifestyle and disease management interventions have noted that 46% of participants abandon the program after a single log-in [46] and high-frequency use of progress-tracking features by returning users [38]. In general, interventions in which information is tailored hold more user interest and show less attrition than those with generic information design [47], an inflexible or static website [48], or ones that give virtual rather than human feedback [38]. A study in which participants with diabetes and/or CVD accessed a web-based portal to view and track information within their EHR noted that those who used data-tracking functions only comprised between 4% and 11% of users and were among the most frequent and consistent users [16]. Clearly, not all EHR-integrated functions will attract all users, and a range of administrative functions may hold value for many patients over clinically oriented functions.

However, within this study, intervention features with EHR-derived data, such as CVD risk score estimation, medications, and risk factor status, were visited more frequently in the early follow-up period than in the later follow-up period. Overall log-in attrition contrasted with comparatively high average monthly log-ins by ongoing users, suggesting that they derived some personal value. The constant appeal of tailored progress tracking was perhaps because of more immediate visual feedback for everyday healthier lifestyle behavior. The informational nature of CVD risk estimation and risk factor status may benefit an initial call to action but not persistent revisits if few updates occur in the shorter term, or it may have entirely disinterested some users. Furthermore, few outcome measures were associated with greater log-in frequency. Further research could explore whether ongoing exposure impacts important but less quantifiable cognitive and emotional stages and processes of health behavior change, [49] as this information becomes increasingly important to design of technologies with this intent [50]. Direct interaction with the primary care provider was not designed into this intervention, so its value to EHR-linked innovations in the local context requires further inquiry. In provider-linked portals with disease self-management intent, more frequent contact from a clinician may nudge website engagement rates [51] although other user differences may be important, such as their primary care providers’ *buy-in*, their baseline self-efficacy, or having a new versus long-term diagnosis [47]. Provider endorsement of a digital application as an extrinsic motivator of engagement is hypothesized but requires investigation [41,52].

High delivery fidelity raises the resource-related question of the optimal support requirements for ongoing website engagement. The type of intervention support appears to benefit different exposure measures. Peer and counselor support facilitate longer session length, for example, when offered in interventions for weight, alcohol, and smoking reduction, whereas updated website content and email and/or telephone contact appear important for site revisits [35]. Other triggers of staff-initiated phone contact could prompt website revisits, for example, a defined number of consecutive days without log-in activity, in addition to scheduled follow-up [44]. Patients are receptive to automated revisit reminders via telephone or email but prefer personal feedback from, for example, a nurse about their disease self-monitoring and health behavior activities [36]. Variations in the intended intervention delivery are generally more likely with complex interventions [21] and with foreseeable differences in provider experience and skill [45]. In this study, the characteristics of study personnel were important contextual and quality factors because the study relied largely on non-face-to-face communication to deliver and support the intervention. In such a context, personality, attitude, and perceived expertise may influence user interest and the reliability of program delivery [21,34]. In addition, contact initiated by participants yielded more app-related problem solving, suggesting that ad hoc support is important to be able to access on the participants’ own timeline of website interaction. Flexible rather than fixed staff facilitation may, therefore, be a worthwhile design consideration because different types of users prefer the option of more human involvement for guidance or accountability, whereas others may choose less or require less over time [46].

### Limitations

First, although typical fidelity measures were chosen, quantified, and described to suit the intervention, it is acknowledged that fidelity research requires systematic scoring practices to better enable comparison and replication of like interventions [53]. Furthermore, there were no prescribed usage or adherence goals with which to compare participant subgroups, but it is acknowledged that uniform concepts of adherence in eHealth would assist in understanding which elements and in what dose, are associated with the intended outcomes of the intervention. [54]. Second, the duration of session and screen visits would have enabled further quantification of use intensity and the relative appeal of features and website components. Third, this study intentionally focused on process rather than outcomes and excluded any assessment of participants’ offline lifestyle behavior for CVD risk factor control. For this reason, other types of engagement with the overall program content may have been underestimated [52]. Fourth, this study excludes cost-effectiveness measures that are important when balancing user uptake with the required facilitator resources, software application oversight and maintenance, and health service support of the EHR linkage software. Furthermore, this study was a summative evaluation. Although intervention delivery and monitoring were dynamic and adaptable throughout the RCT, it is acknowledged that formal evaluations at interim time points can be useful if it is possible to make regular or ad hoc modifications to the program.

### Conclusions

A complex eHealth intervention designed for overall self-directed use can be implemented with high delivery fidelity. Personal progress tracking was consistently used; EHR-derived data features were early but not persistent triggers of revisits. This design intent may reflect how consumers use web-based resources in everyday life but makes usage frequency and thus exposure more unpredictable. Hence, mediating influences and support intensity should be factored into future program planning as drivers of reach and uptake. Despite high delivery fidelity, more frequent intervention use was only associated with small, nonstatistically significant improvements in medication adherence; some clinical measures; and lifestyle behaviors after 12 months. In recognition of multifactorial drivers of engagement, a more explicit personal value proposition should target broad user variables, such as motivation, personal relevance, the context of health care provider use, the timing of program exposure, digital literacy, and preference for log-in adherence accountability. In future research, outcomes related to intervention exposure and reach are important to report so as to expand the evidence about the user and system attributes that promote the uptake of health records with disease self-management functions.

## Abbreviations

CONNECT: Consumer Navigation of Electronic Cardiovascular Tools
CVD: cardiovascular disease
eHEALS: eHealth literacy scale
EHR: electronic health record
RCT: randomized controlled trial

## References

[1] Joseph P, Leong D, McKee M, Anand SS, Schwalm J, Teo K, Mente A, Yusuf S (2017). Reducing the Global Burden of Cardiovascular Disease, Part 1: The Epidemiology and Risk Factors. Circ Res.

[2] Jennings C, Astin F (2017). A multidisciplinary approach to prevention. Eur J Prev Cardiol.

[3] Riegel B, Moser DK, Buck HG, Dickson VV, Dunbar SB, Lee CS, Lennie TA, Lindenfeld J, Mitchell JE, Treat-Jacobson DJ, Webber DE, American Heart Association Council on CardiovascularStroke Nursing; Council on Peripheral Vascular Disease;Council on Quality of CareOutcomes Research (2017). Self-Care for the prevention and management of cardiovascular disease and stroke: a scientific statement for healthcare professionals from the American Heart Association. J Am Heart Assoc.

[4] Barello S, Triberti S, Graffigna G, Libreri C, Serino S, Hibbard J, Riva G (2015). eHealth for patient engagement: a systematic review. Front Psychol.

[5] Fuster V, Kelly BB, Vedanthan R (2011). Promoting global cardiovascular health: moving forward. Circulation.

[6] Pietrzak E, Cotea C, Pullman S (2014). Primary and secondary prevention of cardiovascular disease: is there a place for Internet-based interventions?. J Cardiopulm Rehabil Prev.

[7] Storm V, Dörenkämper J, Reinwand DA, Wienert J, De Vries H, Lippke S (2016). Effectiveness of a web-based computer-tailored multiple-lifestyle intervention for people interested in reducing their cardiovascular risk: a randomized controlled trial. J Med Internet Res.

[8] Devi R, Singh SJ, Powell J, Fulton EA, Igbinedion E, Rees K (2015). Internet-based interventions for the secondary prevention of coronary heart disease. Cochrane Database Syst Rev.

[9] Wantland DJ, Portillo CJ, Holzemer WL, Slaughter R, McGhee EM (2004). The effectiveness of web-based vs non-web-based interventions: a meta-analysis of behavioral change outcomes. J Med Internet Res.

[10] Lie SS, Karlsen B, Oord ER, Graue M, Oftedal B (2017). Dropout from an eHealth intervention for adults with type 2 diabetes: a qualitative study. J Med Internet Res.

[11] Beleigoli AM, Andrade AQ, Cançado AG, Paulo MN, Diniz MD, Ribeiro AL (2019). Web-based digital health interventions for weight loss and lifestyle habit changes in overweight and obese adults: systematic review and meta-analysis. J Med Internet Res.

[12] Donkin L, Christensen H, Naismith SL, Neal B, Hickie IB, Glozier N (2011). A systematic review of the impact of adherence on the effectiveness of e-therapies. J Med Internet Res.

[13] Moore GF, Audrey S, Barker M, Bond L, Bonell C, Hardeman W, Moore L, O'Cathain A, Tinati T, Wight D, Baird J (2015). Process evaluation of complex interventions: medical research council guidance. Br Med J.

[14] Short C, Rebar A, Plotnikoff R, Vandelanotte C (2015). Designing engaging online behaviour change interventions: a proposed model of user engagement. Eur Health Psychol.

[15] Perski O, Blandford A, West R, Michie S (2016). Conceptualising engagement with digital behaviour change interventions: a systematic review using principles from critical interpretive synthesis. Transl Behav Med.

[16] Jones JB, Weiner JP, Shah NR, Stewart WF (2015). The wired patient: patterns of electronic patient portal use among patients with cardiac disease or diabetes. J Med Internet Res.

[17] Irizarry T, DeVito DA, Curran CR (2015). Patient portals and patient engagement: a state of the science review. J Med Internet Res.

[18] Zvoch K (2012). How does fidelity of implementation matter? Using multilevel models to detect relationships between participant outcomes and the delivery and receipt of treatment. Am J Eval.

[19] Hasson H (2010). Systematic evaluation of implementation fidelity of complex interventions in health and social care. Implement Sci.

[20] Holliday LR (2014). Using logic model mapping to evaluate program fidelity. Stud Edu Eval.

[21] Carroll C, Patterson M, Wood S, Booth A, Rick J, Balain S (2007). A conceptual framework for implementation fidelity. Implement Sci.

[22] Oakley A, Strange V, Bonell C, Allen E, Stephenson J (2006). Process evaluation in randomised controlled trials of complex interventions. Br Med J.

[23] Redfern J, Usherwood T, Harris MF, Rodgers A, Hayman N, Panaretto K, Chow C, Lau AYS, Neubeck L, Coorey G, Hersch F, Heeley E, Patel A, Jan S, Zwar N, Peiris D (2014). A randomised controlled trial of a consumer-focused e-health strategy for cardiovascular risk management in primary care: the Consumer Navigation of Electronic Cardiovascular Tools (CONNECT) study protocol. BMJ Open.

[24] Coorey GM, Neubeck L, Usherwood T, Peiris D, Parker S, Lau AYS, Chow C, Panaretto K, Harris M, Zwar N, Redfern J (2017). Implementation of a consumer-focused eHealth intervention for people with moderate-to-high cardiovascular disease risk: protocol for a mixed-methods process evaluation. BMJ Open.

[25] Coorey G, Peiris D, Usherwood T, Neubeck L, Mulley J, Redfern J (2019). Persuasive design features within a consumer-focused eHealth intervention integrated with the electronic health record: A mixed methods study of effectiveness and acceptability. PLoS One.

[26] Coorey G, Peiris D, Neubeck L, Redfern J (2020). A realist evaluation approach to explaining the role of context in the impact of a complex eHealth intervention for improving prevention of cardiovascular disease. BMC Health Serv Res.

[27] Redfern J, Coorey G, Mulley J, Scaria A, Neubeck L, Hafiz N, Pitt C, Weir K, Forbes J, Parker S, Bampi F, Coenen A, Enright G, Wong A, Nguyen T, Harris M, Zwar N, Chow CK, Rodgers A, Heeley E, Panaretto K, Lau A, Hayman N, Usherwood T, Peiris D (2020). A digital health intervention for cardiovascular disease management in primary care (CONNECT) randomized controlled trial. NPJ Digit Med.

[28] Neubeck L, Coorey G, Peiris D, Mulley J, Heeley E, Hersch F, Redfern J (2016). Development of an integrated e-health tool for people with, or at high risk of, cardiovascular disease: The Consumer Navigation of Electronic Cardiovascular Tools (CONNECT) web application. Int J Med Inform.

[29] Redfern J, Thiagalingam A, Jan S, Whittaker R, Hackett ML, Mooney J, De KL, Hillis GS, Chow CK (2014). Development of a set of mobile phone text messages designed for prevention of recurrent cardiovascular events. Eur J Prev Cardiol.

[30] Steckler A, Linnan L (2002). (Eds) Process Evaluation for Public Health Interventions and Research.

[31] Braun V, Clarke V (2006). Using thematic analysis in psychology. Qualitative Research in Psychology.

[32] Australian Bureau of Statistics Estimates of Aboriginal and Torres Strait Islander Australians.

[33] Gearing RE, El-Bassel N, Ghesquiere A, Baldwin S, Gillies J, Ngeow E (2011). Major ingredients of fidelity: a review and scientific guide to improving quality of intervention research implementation. Clin Psychol Rev.

[34] Century J, Rudnick M, Freeman C (2010). A framework for measuring fidelity of implementation: a foundation for shared language and accumulation of knowledge. Am J Eval.

[35] Brouwer W, Kroeze W, Crutzen R, de Nooijer J, de Vries NK, Brug J, Oenema A (2011). Which intervention characteristics are related to more exposure to internet-delivered healthy lifestyle promotion interventions? A systematic review. J Med Internet Res.

[36] Nijland N, van Gemert-Pijnen JE, Kelders SM, Brandenburg BJ, Seydel ER (2011). Factors influencing the use of a Web-based application for supporting the self-care of patients with type 2 diabetes: a longitudinal study. J Med Internet Res.

[37] Tsai R, Bell EJ, Woo H, Baldwin K, Pfeffer MA (2019). How patients use a patient portal: an institutional case study of demographics and usage patterns. Appl Clin Inform.

[38] Glasgow RE, Christiansen SM, Kurz D, King DK, Woolley T, Faber AJ, Estabrooks PA, Strycker L, Toobert D, Dickman J (2011). Engagement in a diabetes self-management website: usage patterns and generalizability of program use. J Med Internet Res.

[39] O'Brien HL, Toms EG (2008). What is user engagement? A conceptual framework for defining user engagement with technology. J Am Soc Inf Sci.

[40] Simblett S, Greer B, Matcham F, Curtis H, Polhemus A, Ferrão J, Gamble P, Wykes T (2018). Barriers to and facilitators of engagement with remote measurement technology for managing health: systematic review and content analysis of findings. J Med Internet Res.

[41] Rogers E (1995). Diffusion of Innovations. 4th edition.

[42] Zvoch K (2009). Treatment fidelity in multisite evaluation: a multilevel longitudinal examination of provider adherence status and change. Am J Eval.

[43] Greenhalgh T, Russell J (2010). Why do evaluations of eHealth programs fail? An alternative set of guiding principles. PLoS Med.

[44] O'Connor S, Hanlon P, O'Donnell CA, Garcia S, Glanville J, Mair FS (2016). Understanding factors affecting patient and public engagement and recruitment to digital health interventions: a systematic review of qualitative studies. BMC Med Inform Decis Mak.

[45] Fraccaro P, Vigo M, Balatsoukas P, Buchan IE, Peek N, van der Veer SN (2018). The influence of patient portals on users' decision making is insufficiently investigated: a systematic methodological review. Int J Med Inform.

[46] van Vugt M, de Wit M, Sieverink F, Roelofsen Y, Hendriks SH, Bilo HJG, Snoek FJ (2016). Uptake and effects of the e-Vita personal health record with self-management support and coaching, for type 2 diabetes patients treated in primary care. J Diabetes Res.

[47] Aalbers T, Baars MA, Rikkert MG (2011). Characteristics of effective internet-mediated interventions to change lifestyle in people aged 50 and older: a systematic review. Ageing Res Rev.

[48] Ludden GD, van Rompay TJ, Kelders SM, van Gemert-Pijnen JE (2015). How to increase reach and adherence of web-based interventions: a design research viewpoint. J Med Internet Res.

[49] Norcross JC, Krebs PM, Prochaska JO (2011). Stages of change. J Clin Psychol.

[50] Mohr DC, Schueller SM, Montague E, Burns MN, Rashidi P (2014). The behavioral intervention technology model: an integrated conceptual and technological framework for eHealth and mHealth interventions. J Med Internet Res.

[51] Tate DF, Jackvony EH, Wing RR (2003). Effects of internet behavioral counseling on weight loss in adults at risk for type 2 diabetes: a randomized trial. J Am Med Assoc.

[52] Yardley L, Spring BJ, Riper H, Morrison LG, Crane DH, Curtis K, Merchant GC, Naughton F, Blandford A (2016). Understanding and promoting effective engagement with digital behavior change interventions. Am J Prev Med.

[53] Slaughter SE, Hill JN, Snelgrove-Clarke E (2015). What is the extent and quality of documentation and reporting of fidelity to implementation strategies: a scoping review. Implement Sci.

[54] Sieverink F, Kelders SM, van Gemert-Pijnen E (2017). Clarifying the concept of adherence to eHealth technology: systematic review on when usage becomes adherence. J Med Internet Res.

